# The impact of the mathematical adversity quotient on preparatory students’ mathematics learning engagement: moderated mediation effect analysis

**DOI:** 10.3389/fpsyg.2026.1725090

**Published:** 2026-03-19

**Authors:** Juanjuan Liu, Yu Sun, Yatao Li

**Affiliations:** 1 Faculty of Education, Yunnan Normal University, Kunming, China; 2 College of Preparatory Education, Yunnan Minzu University, Kunming, China

**Keywords:** math anxiety, mathematical adversity quotient, mathematics learning engagement, preparatory students, teacher support

## Abstract

This study investigated how preparatory students’ engagement in mathematics emerges from the interplay between internal resources, emotional states, and the classroom context. A sample of 484 preparatory students completed the Mathematical Adversity Quotient Scale, Math Anxiety Scale, Teacher Support Scale, and Mathematics Learning Engagement Scale. Using PROCESS (Model 4 and Model 15), we tested a moderated mediation framework in which math anxiety mediates the effect of the Mathematical Adversity Quotient (MAQ) on engagement, and teacher support moderates the MAQ → mathematics learning engagement and math anxiety → mathematics learning engagement paths. The results indicated that MAQ positively predicted mathematics engagement both directly and indirectly via reduced math anxiety. Teacher support exhibited a double-edged moderating role: it amplified the positive association between MAQ and engagement, yet intensified the negative association between math anxiety and engagement at higher levels of support. These findings refine the person–environment framework of learning engagement by demonstrating that teacher support can simultaneously amplify the positive influence of internal coping resources while intensifying the negative impact of math anxiety on engagement. Practically, preparatory mathematics programs should focus on developing students’ MAQ, shift from universal to diagnostic support that prioritizes psychological safety for highly anxious students, and take advantage of the transitional nature of the preparatory stage by strengthening resilience alongside knowledge remediation.

## 1 Introduction

Education serves as a strategic cornerstone of national development and has the historical responsibility of cultivating high-quality professional talents. As a unified multi-ethnic country, China’s ethnic minority education is an integral component of its national education system. Its distinctiveness lies in preserving the inheritance of cultural diversity and promoting regional education equity (Zhao and Yang, 2022). In recent years, the state has maintained its momentum in strengthening ethnic education by implementing specific policies. Preparatory education, which serves as a key transition mechanism connecting basic education and higher education, has the core function of optimizing ethnic talent reserves and promoting equitable educational opportunities from the outset. The academic performance of preparatory students influences not only individual career development but also directly affects the overall quality of ethnic education and the success of regional coordinated development. Preparatory education, therefore, is not merely about transmitting knowledge but also about cultivating higher-order thinking skills and intellectual resilience (Vygotsky, 1978; Heckman and Kautz, 2013; Duckworth et al., 2007). Mathematics plays a pivotal role among academic subjects due to its foundational role and its capacity to foster logical thinking, abstract reasoning, and problem-solving abilities (Goldin, 2008; Boaler, 2022). However, despite the central role of mathematics in education and development, many preparatory students face significant challenges in learning the subject. This challenge is particularly pronounced in ethnic regions, where the difficulty of the subject matter is compounded by the multiple burdens stemming from language barriers, cultural differences, and uneven resource allocation. These factors make students’ commitment to mathematics learning more susceptible to disruption from both emotional and environmental influences.

Preparatory students are ethnic minority students who meet specific criteria. After participating in the National College Entrance Examination and benefiting from certain score reduction policies, they are admitted into state-designated higher education institutions based on merit. These students undergo 1 to 2 years of preparatory studies (Zheng, 2019). As a distinct and crucial cohort within higher education, preparatory students are navigating a vital transition phase from secondary school to university. Most preparatory students come from ethnic minority regions, which often face a shortage of educational resources—particularly at the basic education level. Variations in the quality of mathematics teaching result in some students entering college and university with a weak mathematics foundation, meaning they face challenges in adapting to the learning requirements of advanced mathematics courses (Faulkner et al., 2014). Furthermore, in some ethnic regions, students use their own ethnic language as their mother tongue; the learning barrier of Chinese as a second language further increases their difficulty in understanding mathematical terms and concepts, thereby reducing their engagement in mathematics learning (Barwell, 2005; Moschkovich, 2010; Setati and Adler, 2000). Therefore, enhancing preparatory students’ engagement in mathematics learning and supporting them in overcoming difficulties has become a critical issue for improving their academic performance and optimizing the quality of mathematics education (Fredricks et al., 2004; Jansen and Middleton, 2011; Boaler, 2022). Accordingly, we propose a “contextual amplification” effect in the preparatory setting: heightened linguistic load and heterogeneous prior mathematics preparation intensify threat appraisals, thereby amplifying the impact of anxiety and the importance of supportive resources on student engagement.

Mathematics learning engagement encompasses the level of students’ active participation and the depth of their emotional experience during mathematics learning (Kong et al., 2003). It serves as a key indicator of learning quality, as well as playing a crucial role in shaping students’ academic achievement in mathematics (Fredricks et al., 2004; Skinner et al., 2009). Research has shown that mathematics learning engagement is influenced by many factors, including the quality of teacher–student interaction (Rimm-Kaufman et al., 2015), learning interest (Wu et al., 2022), academic self-efficacy (Ozkal, 2019), and parents’ educational expectations and parental involvement (No et al., 2016; Wang and Sheikh-Khalil, 2014). With ongoing educational reforms and the rapid development of artificial intelligence technology, the role of non-intellectual factors in learning has received increasing attention (OECD, 2017). Mathematical Adversity Quotient (MAQ) refers to a domain-specific psychological capacity that reflects how students perceive, interpret, and respond to adversity encountered in mathematics learning. Conceptually grounded in Stoltz (1999) Adversity Quotient framework and adapted to the mathematics learning context (Cao et al., 2015; Amir et al., 2021), MAQ emphasizes individuals’ typical appraisal patterns and coping orientations when facing mathematical difficulties, rather than the frequency of prior failure experiences or the presence of negative attitudes toward mathematics.

In line with the original Adversity Quotient model, MAQ is conceptualized as a multidimensional construct comprising four interrelated dimensions: mathematical control, referring to students’ perceived ability to influence learning outcomes when facing difficulty; mathematical belonging, reflecting students’ perceived attribution of responsibility for mathematical setbacks and their sense of personal involvement in addressing these difficulties; mathematical impact, indicating students’ appraisal of how extensively mathematical difficulties affect their overall learning process; and mathematical extension, representing students’ perceptions of the duration and spillover of mathematical adversity over time, as well as their beliefs about whether such difficulties are temporary and recoverable (Cao et al., 2015).

Importantly, the term “quotient” is used to underscore that MAQ captures a stable yet malleable coping capacity rather than a single performance score or a fixed personality trait. Operationally, MAQ is assessed through self-report items that probe students’ habitual interpretations of mathematical setbacks—such as perceived controllability, attributional responsibility, and beliefs about persistence—rather than their immediate emotional reactions or levels of achievement. Higher MAQ scores therefore indicate a stronger tendency to construe mathematical adversity as manageable, temporary, and open to improvement.

From a functional perspective, MAQ represents an important individual resource that supports mathematics learning engagement through multiple psychological pathways. Drawing on self-regulated learning theory and social cognitive theory, students with higher MAQ are more likely to regulate negative emotions elicited by difficulty, maintain adaptive control beliefs, and persist in strategic effort under challenge (Bandura et al., 1997; Zimmerman, 2013; Zimmerman and Schunk, 2013). In this sense, MAQ shares conceptual overlap with domain-specific academic resilience, while retaining a distinct emphasis on adversity appraisal processes derived from the Adversity Quotient framework (Duckworth et al., 2007; Heckman and Kautz, 2013). Conversely, students with lower MAQ are more vulnerable to maladaptive attributions and learned helplessness tendencies, which may undermine sustained engagement in mathematics learning when difficulties arise.

In studying factors affecting mathematics learning engagement, researchers have increasingly emphasized the central role of academic emotions (Qi et al., 2024). From a person–environment perspective, academic emotions serve as proximal regulatory mechanisms through which internal resources and contextual experiences shape students’ behavioral, cognitive, and affective engagement. Recent research has highlighted the importance of internal regulatory capacities in this process. For example, Sorrenti et al. (2025) found that students’ awareness of present experiences negatively predicted emotional problems, which in turn were associated with higher levels of school refusal. Although their study focused on school refusal rather than learning engagement directly, it demonstrates how deficits in internal awareness operate through emotional dysregulation to undermine adaptive school functioning. Together, these findings support the view that internal appraisal and regulatory systems constitute foundational mechanisms linking emotional processes to variations in students’ engagement-related outcomes.

In mathematics learning contexts, math anxiety represents a domain-specific manifestation of such emotional dysregulation. As an achievement emotion closely tied to perceived control and task value (Pekrun, 2006), math anxiety emerges when students appraise mathematical demands as threatening or uncontrollable. Existing studies consistently show that math anxiety negatively predicts mathematics learning engagement (Barroso et al., 2021; Daker et al., 2021; Guo and Liao, 2025; Quintero et al., 2022; Wang, 2020). Math anxiety depletes working memory resources and disrupts attentional control (Ashcraft and Krause, 2007). According to cognitive interference theory, anxiety triggers task-irrelevant thoughts such as worry and self-evaluative rumination (Sarason, 1984; Wine, 1971). These intrusive cognitions consume executive resources required for problem-solving, thereby reducing processing efficiency (Eysenck and Calvo, 1992; Eysenck et al., 2007). In mathematics tasks, such interference functions as an internal “secondary task,” undermining persistence and sustained engagement even when students attempt to remain on task. Thus, academic emotions—particularly anxiety—operate as immediate psychological pathways through which internal appraisals translate into variations in engagement. Within this person–environment framework, math anxiety therefore serves as the pivotal emotional mechanism through which internal adversity appraisals are translated into variations in mathematics learning engagement.

Importantly, individual differences in internal coping resources—such as the MAQ—may shape how students appraise mathematical challenges in the first place. Students with higher MAQ are more likely to interpret difficulties as manageable rather than threatening, thereby reducing anxiety activation and protecting engagement. Conversely, lower MAQ may intensify threat appraisal, increasing emotional dysregulation and subsequent withdrawal from learning tasks. In this sense, MAQ may influence engagement indirectly by regulating the emotional processes that arise from students’ cognitive evaluations of adversity.

Further research has distinguished between state and trait math anxiety, showing that both forms significantly influence students’ math performance through the mediating role of core executive functions such as attention control and working memory. The findings highlight how anxiety-induced cognitive interference can lead to decreased engagement in learning (Orbach et al., 2020). For preparatory students, math anxiety may be more pronounced due to their specific linguistic and cultural backgrounds. This not only exacerbates their fear of math learning but also further weakens their self-confidence and academic performance, leading to decreased engagement in math learning.

Studies have also confirmed a close relationship between MAQ and math anxiety (Wahyuningtyas et al., 2020). Math anxiety has been widely recognized as a multidimensional concept that manifests in different stages of the examination process—before, during, and after the examination. Academic emotions such as anxiety fluctuate at various stages and have different effects on students’ learning processes and outcomes (Pekrun et al., 2002; Szczygieł and Pieronkiewicz, 2021). Pre-exam anxiety is often associated with anticipatory worry and negative expectations, which may hinder students’ cognitive preparation. On the other hand, anxiety during the test interferes with working memory and attention control, directly impairing task performance. Post-test anxiety is often associated with emotional rumination and regret, which may affect students’ future learning motivation. These different types of anxiety are not only harmful to academic performance but also significantly affect students’ MAQ. Synthesizing these accounts, students with lower MAQ are more likely to perceive mathematical challenges as threatening, which triggers stronger anxiety and greater cognitive interference; this interference diminishes sustained attention, persistence, and strategic effort—key components of learning engagement—thus positioning math anxiety as a plausible mediator linking MAQ to engagement. Therefore, the study puts forward,

*Hypothesis* 1: Math anxiety serves as a mediating factor in the relationship between the Mathematical Adversity Quotient and students’ engagement in mathematics learning.

In sum, the proposed model conceptualizes MAQ as an internal coping resource, math anxiety as the emotional mechanism linking cognitive appraisals to engagement, and teacher support as a contextual regulator within a person–environment framework. In this system, students’ interpretations of mathematical challenges shape emotional responses—particularly anxiety—which in turn determine the degree of cognitive, behavioral, and affective engagement. Teacher support conditions how these internal and emotional processes translate into engagement outcomes.

Within this framework, the influence of MAQ on engagement cannot be understood in isolation, as internal coping resources are enacted within specific classroom contexts rather than operating independently. This perspective aligns with broader ecological approaches to academic functioning. Using PISA 2022 data, Wei and Zhang (2025) applied a multi-layer ecological framework and found that psychological variables (including math anxiety) and school-related factors independently contributed to mathematics performance. Their findings highlight the necessity of examining emotional processes alongside contextual resources when explaining academic functioning. Accordingly, although MAQ reflects students’ internal resilience in facing mathematical adversity, its impact on engagement may depend on the classroom environment in which learning unfolds.

Among contextual factors, teacher support represents one of the most proximal and influential classroom resources. As well as being knowledge transmitters, teachers provide emotional, autonomy, and cognitive support that shape students’ appraisals of academic demands, particularly in shaping students’ emotional responses to mathematical challenges. Existing research has confirmed that teacher support positively influences students’ emotional development, cognitive growth, and academic achievement (Lei et al., 2018; Sun et al., 2021; Szczygieł, 2020a; Xu, 2024; Zhang and Hu, 2025). From a regulatory perspective, teacher support may strengthen or weaken the impact of internal coping resources on academic emotions. When students face learning difficulties, timely guidance and supportive feedback can enhance psychological resilience and reduce threat appraisals. For students with high MAQ, teacher support may function as strategic scaffolding, amplifying their adaptive interpretations of difficulty and further enhancing engagement (Belland et al., 2013). Conversely, for students with low MAQ, emotional reassurance and autonomy-supportive practices may be particularly critical in buffering anxiety activation and preventing disengagement (Kaskens et al., 2020; Leahy, 2012). Therefore, teacher support may function not only as a direct predictor of engagement but also as a contextual regulator that shapes how internal coping resources translate into emotional experiences and, ultimately, engagement outcomes.

Empirical research provides strong evidence for the mechanisms underlying the role of teacher support. Longitudinal studies by Li et al. (2021, 2023) indicate that teacher support significantly enhances students’ learning engagement across behavioral, cognitive, and emotional dimensions, as well as indirectly influencing learning performance by improving attitudes toward mathematics. Notably, the results of latent profile analysis show that students in the lowest teacher support group exhibit the weakest mathematical engagement and higher anxiety levels. These findings align with the core tenets of control value theory, which posits that teacher support influences academic emotions and learning engagement by moderating students’ sense of control and the value they place on tasks (Broda et al., 2023; Wang et al., 2024). Based on the aforementioned theoretical analysis and empirical evidence, this study proposes,

*Hypothesis* 2: Teacher support moderates the direct association between the Mathematical Adversity Quotient and mathematics learning engagement.

Furthermore, math anxiety, as an affective impairment variable contrasting with MAQ, significantly depletes students’ cognitive resources and triggers avoidance behaviors (Ashcraft and Krause, 2007; Hembree, 1990). The stress buffering model of social support (Cohen and Wills, 1985) provides a crucial theoretical perspective for this influence pathway. This model suggests that teacher support, as a proximal social resource, can effectively mitigate the negative impact of anxiety as an academic stressor on learning behavior by providing emotional security, breaking down task difficulty, and reshaping positive beliefs. It helps students to reassess threats, transforming anxiety-induced feelings of helplessness into manageable challenges and thereby protecting their learning engagement. Therefore, this study proposes,

*Hypothesis* 3: Teacher support moderates the effect of math anxiety on mathematics learning engagement.

In summary, the current research on MAQ, math anxiety, and mathematics learning engagement is mainly concentrated in middle school and primary school settings, while research on preparatory students, especially preparatory students in ethnic minority areas, is relatively lacking. Previous studies have mostly focused solely on the direct impact of math anxiety on mathematics learning engagement, with limited research exploring the underlying mechanisms through which MAQ influences mathematics learning engagement. In addition, although teacher support has been found to have a significant impact on mathematics learning engagement, the mechanism of how MAQ is regulated by teacher support has not been fully studied. Based on this, this study will focus on preparatory students and explore the interaction between MAQ, math anxiety, and teacher support. The aim is to provide theoretical support and practical guidance for preparatory students to overcome difficulties in math learning and improve mathematics learning engagement and academic performance.

## 2 Materials and methods

### 2.1 Participants

A total of 502 preparatory students were selected through a combination of whole-group and random sampling. After excluding 18 invalid questionnaires, 484 valid responses were obtained, resulting in a valid response rate of 96.41%. Among the participants, 158 were male (32.64%) and 326 were female (67.36%). 207 students were in liberal arts (42.77%) programs and 277 (57.23%) were in science programs.

### 2.2 Measures

#### 2.2.1 Mathematical adversity quotient scale

The Mathematical Adversity Quotient Scale (MAQS) was adapted from the instrument developed by Cao et al. (2015), who conceptualized mathematical adversity within the framework of Stoltz (1999) Adversity Quotient theory. The scale comprises four dimensions: mathematical control, mathematical belonging, mathematical impact, and mathematical extension, totaling 20 items. Each item presents a concrete mathematics-related challenge (e.g., difficulty understanding a lesson, encountering unsolved homework problems, or perceiving mathematics as difficult) and asks students to evaluate their perceived controllability, responsibility, impact, or persistence of the setback. Responses were rated on a 5-point scale tailored to each scenario (e.g., from “completely able to improve” to “impossible to change,” or from “almost no impact” to “affects all aspects of life”), with higher scores indicating stronger mathematical adversity coping capacity (*α* = 0.95).

#### 2.2.2 Math anxiety scale

Adapted from Plake and Parker’s MARS-R (Plake and Parker, 1982), this scale comprises three dimensions: mathematics test task anxiety, mathematics classroom anxiety, and mathematics personal anxiety, totaling 10 items. Sample items include: “When you do a difficult math problem” and “When you think about an upcoming mathematics final exam.” Participants rated their level of anxiety on a 5-point scale (1 = no anxiety to 5 = very high anxiety), with higher scores indicating greater math anxiety (*α* = 0.93).

#### 2.2.3 Teacher support scale

The 17-item Teacher Support Scale (TSS) evaluates perceived emotional support, autonomy support, and cognitive support (Chai and Gong, 2013). Emotional support items reflect teachers’ affirmation, encouragement, and relational care (e.g., “My mathematics teacher holds a positive attitude toward my mathematics learning performance” and “My teacher encourages and affirms my efforts in learning mathematics”). Autonomy support items capture opportunities for student voice and independent thinking (e.g., “My teacher listens to our different solution strategies,” “My teacher considers our opinions to adjust instruction,” and “My teacher leaves sufficient time for independent thinking in class”). Instructional support items reflect structured guidance and cognitive scaffolding (e.g., “My teacher guides us patiently when solving problems rather than giving answers too quickly” and “Homework includes both foundational practice and advanced tasks with appropriate difficulty”). Responses were rated on a 5-point Likert scale (1 = strongly disagree to 5 = strongly agree), with higher scores indicating greater perceived teacher support (*α* = 0.96).

#### 2.2.4 Mathematics learning engagement scale

The Mathematics Learning Engagement Scale (MLES), adapted from the Utrecht Work Engagement Scale for Students (UWES-S; Schaufeli and Bakker, 2003), consists of 17 items assessing students’ engagement in mathematics learning across three dimensions: vigor, dedication, and absorption. Example items include: “I work very hard in mathematics learning” (vigor), “I find solving mathematics problems interesting” (dedication), and “I listen attentively during mathematics class” (absorption). Reverse-coded items included statements such as “I often think about other things during mathematics class,” which were recoded prior to analysis. Responses were rated on a 5-point Likert scale (1 = very inconsistent with me to 5 = very consistent with me). Higher scores indicate greater mathematics learning engagement (α = 0.89).

### 2.3 Procedure

This study strictly adhered to the internationally recognized Declaration of Helsinki. Prior to data collection, informed consent was obtained from all participants on a completely voluntary basis. The questionnaires were administered in a fully anonymous manner, and no information that could directly or indirectly identify the participants was collected. This study received ethical approval from the Institutional Review Board (Date: May 13, 2025, Ethic Code: FEIRB20250036).

### 2.4 Statistical analyses

This study employed SPSS 26.0 for data processing and statistical analysis. Descriptive statistics and correlation analysis were first conducted on the collected data. Subsequently, independent samples t-tests were used to examine differences in MAQ, math anxiety, teacher support, and mathematics learning engagement across gender and academic disciplines. Finally, Hayes and Matthes (2009) PROCESS Model 4 and Model 15 were applied to conduct mediation effect analysis and test for moderated mediation effects, respectively.

## 3 Results

### 3.1 Descriptive statistics and correlation analysis of variables

Table 1 shows the descriptive statistics and correlation analysis results of each variable. The correlation analysis revealed that MAQ was significantly negatively associated with math anxiety (*r* = −0.59, *p* < 0.01) and significantly positively associated with both teacher support (*r* = 0.37, *p* < 0.01) and mathematics learning engagement (*r* = 0.44, *p* < 0.01). Math anxiety showed significant negative correlations with teacher support (*r* = −0.27, *p* < 0.01) and mathematics learning engagement (*r* = −0.49, *p* < 0.01). Additionally, teacher support was positively correlated with mathematics learning engagement (*r* = 0.47, *p* < 0.01).

**Table 1:** Descriptive statistics, skewness, kurtosis, and correlations.

Variable	M	SD	Skewness	Kurtosis	Correlation			
					1	2	3	4
1. Mathematical Adversity Quotient	3.26	0.69	0.24	−0.51	1	-	-	-
2. Math anxiety	2.47	0.92	0.50	−0.26	−0.58**	1	-	-
3. Teacher support	3.63	0.73	0.01	−0.14	0.36**	−0.27**	1	-
4. Mathematics learning engagement	3.06	0.55	0.56	1.55	0.42**	−0.49**	0.47**	1

M, mean; SD, standard deviations; **p < 0.001.

### 3.2 Tests of variance for each variable

An independent samples t-test was conducted to examine gender and subject category differences in MAQ, math anxiety, teacher support, and mathematics learning engagement among preparatory students; the results are presented in Table 2. The findings revealed significant differences in MAQ based on both gender and subject category. Specifically, male students demonstrated significantly higher MAQ scores than female students, and science students outperformed liberal arts students in overall MAQ. Significant gender and subject-related differences were also observed in math anxiety: female and liberal arts students reported notably higher anxiety levels than male and science students. In contrast, teacher support did not vary significantly by gender or subject category. Mathematics learning engagement showed no significant gender difference but did differ by category, with science students reporting greater engagement than liberal arts students.

**Table 2:** A test of differences in MAQ, math anxiety, teacher support, and mathematics learning engagement across demographic variables.

Variable		Mathematical adversity quotient	Math anxiety	Teacher support	Mathematics learning engagement
Gender	Male (*n* = 158)	3.46 ± 0.70	2.32 ± 0.95	3.65 ± 0.79	3.10 ± 0.66
	Female (*n* = 326)	3.16 ± 0.67	2.55 ± 0.90	3.62 ± 0.69	3.04 ± 0.49
	t	4.42***	−2.55*	0.34	1.02
	Cohen’s d	0.44	−0.25	0.03	0.10
Subject	Liberal arts students (*n* = 207)	3.06 ± 0.64	2.81 ± 0.94	3.58 ± 0.69	2.93 ± 0.51
	Science students (*n* = 277)	3.41 ± 0.69	2.22 ± 0.82	3.67 ± 0.75	3.16 ± 0.56
	t	−5.75***	7.25***	−1.42	−4.63***
	Cohen’s d	0.52	0.68	−0.13	−0.43

*p < 0.05; **p < 0.01; ***p < 0.001.

### 3.3 Test of moderated mediation model

Model 4 of the SPSS macro program PROCESS was used to examine the mediating role of math anxiety between MAQ and mathematics learning engagement. The results are shown in Table 3. After controlling for gender and subject, MAQ significantly predicted mathematics learning engagement (*β* = 0.32, *p* < 0.001). Even after incorporating mediating variables, the direct predictive effect of MAQ on mathematics learning engagement remained significant (*β* = 0.17, *p* < 0.001). MAQ also significantly negatively predicted math anxiety (*β* = −0.71, *p* < 0.001), and math anxiety also negatively predicted mathematics learning engagement (*β* = −0.21, *p* < 0.001). The nonparametric percentile bootstrap test for bias correction revealed that math anxiety significantly mediated the relationship between MAQ and mathematics learning engagement (95% confidence interval did not include 0), explaining 46.88% of the mediating effect (Table 4).

**Table 3:** Mediation effect test.

Outcome variable	Predictor variable	R^2^	F	coeff	se	t
Mathematics learning engagement	Mathematical Adversity Quotient	0.19	37.00	0.32	0.03	9.25***
Math anxiety	Mathematical Adversity Quotient	0.36	91.23	−0.71	0.05	−13.91***
Mathematics learning engagement	Mathematical Adversity Quotient	0.27	44.15	0.17	0.04	4.31***
	Math anxiety			−0.21	0.03	−7.71***

***p < 0.001.

**Table 4:** Proportion of mediating effect.

Effect Type	Effect	BootSE	LLCI	ULCI	Proportion of effect
Total effect	0.32	0.03	0.25	0.38	
Direct effect	0.17	0.04	0.09	0.24	53.12%
Indirect effect	0.15	0.02	0.11	0.20	46.88%

**Figure 1:**
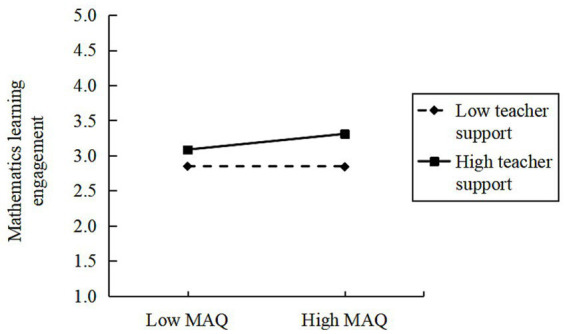
Moderating effect of teacher support between MAQ and mathematics learning engagement.

**Figure 2:**
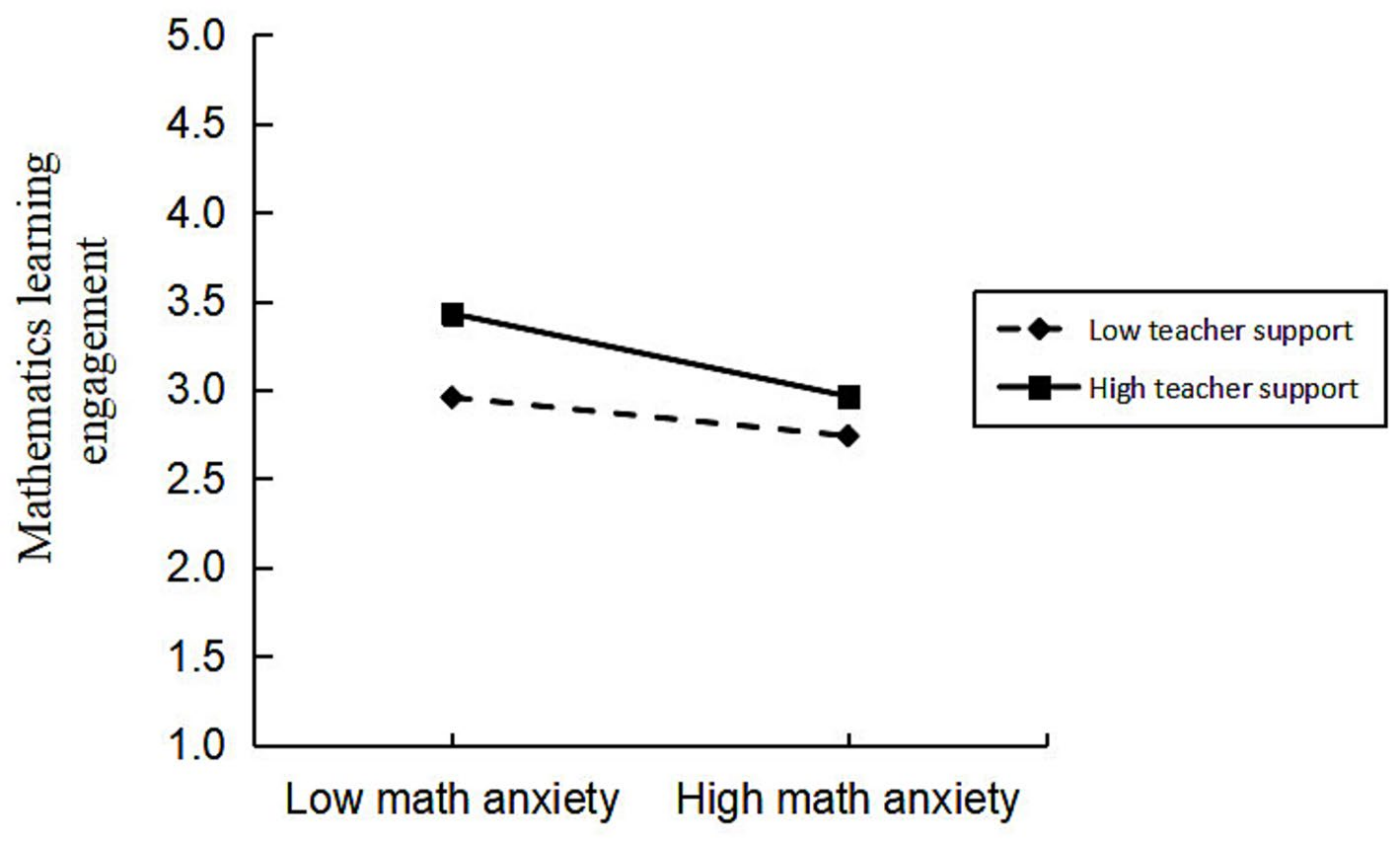
Moderating effect of teacher support between math anxiety and mathematics learning engagement.

Subsequently, we employed Model 15 from the SPSS macro PROCESS to examine the moderating effect of teacher support; the results are presented in Table 5. After controlling for gender and subject factors, the interaction term “Mathematical Adversity Quotient × teacher support” significantly predicted mathematics learning engagement (*β* = 0.12, *p* < 0.01), while the interaction term “math anxiety × teacher support” also significantly predicted mathematics learning engagement (*β* = −0.09, *p* < 0.01). This indicates that teacher support not only moderates the direct effect of MAQ on mathematics learning engagement but also moderates the impact of math anxiety on mathematics learning engagement. Further simple slope analysis (Figures 1, 2) revealed that when teacher support was low, the predictive effect of MAQ on mathematics learning engagement was insignificant. However, under medium to high levels of teacher support, the positive predictive effect of MAQ on mathematics learning engagement gradually strengthened, exhibiting an increasing trend with rising teacher support. Simultaneously, across all three levels of teacher support, the negative effect of math anxiety on engagement was significant and intensified with increasing teacher support. This indicates that in high-support contexts, students with higher math anxiety experience greater suppression of their mathematics learning engagement.

**Table 5:** Modified mediation model testing.

Outcome variable	Predictor variable	R	R2	F	Coeff	Se	t
Math anxiety	Gender	0.60	0.36	91.23	−0.04	0.07	−0.50
	Subject				−0.35	0.07	−4.95***
	Mathematical Adversity Quotient				−0.71	0.05	−13.91***
Mathematics learning engagement	Gender	0.64	0.42	48.28	0.03	0.04	0.70
	Subject				0.04	0.04	1.04
	Mathematical Adversity Quotient				−0.36	0.16	−2.25*
	Math anxiety				0.15	0.13	1.22
	Teacher support				0.08	0.21	0.39
	Mathematical Adversity Quotient × Teacher support				0.12	0.04	2.72**
	Math anxiety × Teacher support				−0.09	0.03	−2.71**

*p < 0.05; **p < 0.01; ***p < 0.001.

## 4 Discussion

Focusing on preparatory students, this study constructed a moderated mediation model to examine the complex interplay among MAQ, math anxiety, teacher support, and mathematics learning engagement. The findings validated the expected hypotheses and uncovered insightful instructive patterns, which are discussed below in the context of existing theories and research.

The study found that MAQ directly predicts mathematics learning engagement and also exerts an indirect promotional effect by alleviating math anxiety. This mediated pathway validates the core tenets of control value theory (Pekrun, 2006; Pekrun and Perry, 2014). This theory emphasizes that when individuals possess a high sense of control over learning activities (as represented by the adversity quotient, reflecting the ability to cope with setbacks and take proactive control), they can effectively reduce the interference of negative emotions like anxiety, thereby increasing their willingness to engage in learning (Eccles and Wigfield, 2002). Specifically, students with a high MAQ maintain greater confidence and perseverance when encountering mathematical challenges. They perceive task difficulty as less threatening, allowing them to allocate cognitive resources toward the learning process itself rather than expending them on emotional avoidance. This fosters more positive behavioral and affective engagement (Ashcraft and Krause, 2007; Eysenck et al., 2007). This mediating mechanism suggests that MAQ functions not only as a resilience-related psychological trait but also as a regulator of emotional processes. By reducing anxiety-related interference, MAQ helps free cognitive resources for sustained engagement in learning tasks. Therefore, enhancing engagement in mathematics learning should not be confined to behavioral interventions but should extend to cognitive and emotional dimensions, viewing resilience cultivation and anxiety management as interrelated dual pathways (Hembree, 1990; Putwain and Symes, 2011). This mechanism holds particular significance for preparatory students, who commonly exhibit “foundational fragility”—making them more prone to feelings of helplessness when learning new concepts, which in turn heightens math anxiety (Devine et al., 2012). Against this backdrop, MAQ can be conceptualized as a form of psychological capital that encourages students to attribute learning setbacks to temporary and controllable factors rather than fixed ability deficits. Such adaptive attribution patterns may reduce the activation of negative emotions and help sustain motivational investment in mathematics learning (Dweck, 2006; Simms, 2016). Therefore, for preparatory students, enhancing their MAQ is not only about character development but also serves as an emotional protection strategy that addresses their academic challenges. It provides crucial psychological support to bridge foundational gaps and enable effective engagement.

The most theoretically significant finding of this study concerns the “double-edged sword” moderating role of teacher support. Teacher support amplified the positive association between MAQ and mathematics learning engagement. At the same time, however, higher levels of teacher support intensified the negative impact of math anxiety on engagement. These findings indicate that teacher support is not a uniform facilitator; rather, its effectiveness depends on students’ internal psychological states.

This pattern is consistent with the Social Support Gain Hypothesis and self-determination theory. When teachers provide autonomy-supportive and clearly structured guidance, students are more likely to internalize external demands and regulate their learning more effectively, thereby strengthening engagement gains for students with high MAQ (Ryan and Deci, 2000; Jang et al., 2010). However, if support is perceived as controlling or outcome-oriented, highly anxious students may interpret it as a signal of evaluative pressure, which can amplify stress and further suppress engagement (Assor et al., 2002; Putwain and Symes, 2011). From the perspective of attentional control theory (Eysenck et al., 2023), such heightened pressure may increase cognitive interference. Therefore, the orientation and psychological framing of support—rather than its mere quantity—appear critical in determining whether support functions as a protective or risk factor (Reeve, 2006).

Beyond this primary interpretation, an alternative explanation of the moderating role of teacher support also warrants consideration. Rather than focusing on the orientation or quality of support itself, this perspective emphasizes students’ psychological responses to intensified support under conditions of elevated math anxiety. Highly anxious students may become increasingly reliant on external guidance and reassurance, such that greater teacher involvement fosters dependency rather than autonomy. From this viewpoint, intensified support may heighten students’ self-monitoring and sensitivity to evaluation, increasing cognitive load and reinforcing threat appraisals even when support is well intentioned. As a result, teacher support may function as a contextual signal of heightened academic scrutiny rather than emotional safety for anxious students, thereby amplifying anxiety-related disengagement. This dependency-based interpretation highlights that students’ internal emotional states and perceived autonomy critically shape how support is received and utilized, complementing—but not duplicating—explanations centered on support orientation.

It should be noted that, given the correlational design of the present study, these interpretations remain tentative, and longitudinal or experimental research is needed to clarify the directionality of dependency and support effects.

Furthermore, the differential findings of this study align with extensive prior research, indicating that female and liberal arts students tend to exhibit lower MAQ and engagement while displaying higher levels of math anxiety (Carey et al., 2015; Devine et al., 2012; Else-Quest et al., 2010; Szczygieł, 2020b). This phenomenon may be influenced by multiple factors, including sociocultural expectations, gender stereotype threat, and differences in disciplinary thinking patterns (Barroso et al., 2021; Vos et al., 2023; Luo and Chen, 2024). The study findings suggest that preparatory education should address intragroup diversity by adopting a tailored instructional approach in educational interventions. Particular attention should be paid to the emotional experiences of female and arts students in mathematics learning. Targeted resilience training and anxiety management can help them overcome psychological barriers and foster a positive identity in relation to the subject (Wang et al., 2013).

The present findings should also be interpreted in light of the unique characteristics of preparatory students as a transitional population. Preparatory students typically enter higher education with heterogeneous academic foundations, heightened linguistic and cultural demands, and increased sensitivity to evaluation, all of which may intensify threat appraisals and emotional responses in mathematics learning contexts. These features likely amplify the roles of both internal coping resources, such as MAQ, and contextual factors, such as teacher support, in shaping learning engagement.

At the same time, some of the mechanisms identified in this study—particularly the mediating role of math anxiety linking adversity-related coping resources to engagement—may reflect more general psychological processes applicable beyond the preparatory context. However, the strength and configuration of these relationships may be especially pronounced among preparatory students due to their transitional status. Accordingly, caution is warranted in generalizing the magnitude of effects to other student populations, while the underlying theoretical pathways may retain broader relevance.

## 5 Conclusion and limitations

In summary, this study advances understanding of the mechanisms underlying students’ engagement in mathematics learning. The findings suggest that engagement emerges from the dynamic interplay among internal coping resources (MAQ), emotional processes (math anxiety), and contextual factors (teacher support), rather than being determined by isolated personal or environmental variables. The primary theoretical contribution of this study lies in proposing a moderated mediation framework that clarifies how teacher support may function as a “double-edged sword,” strengthening the positive influence of MAQ while potentially intensifying the detrimental effects of anxiety under certain conditions.

From a practical perspective, the findings imply that preparatory mathematics programs may benefit from incorporating structured resilience-building components alongside knowledge remediation. For example, instructional designs could intentionally include manageable challenge segments that encourage adaptive attribution patterns and promote students’ capacity to recover from setbacks (Dweck, 2006). In addition, the results suggest that teacher support practices may need to move from uniform provision toward more diagnostic and differentiated approaches. Teachers’ sensitivity to students’ anxiety levels and perceived pressure may be particularly important, as highly anxious students may require psychologically safe and autonomy-supportive environments that minimize evaluative threat (Assor et al., 2002). More broadly, these findings indicate that preparatory education may benefit from extending beyond knowledge compensation to include systematic attention to resilience development and emotional regulation processes during this transitional stage.

Despite these contributions, several limitations should be acknowledged. First, the sample was drawn from preparatory students in a specific region with relatively homogeneous cultural characteristics, which may limit the generalizability of the findings. Second, the cross-sectional design precludes causal inferences regarding the directionality of the observed relationships. Third, all variables were assessed using self-report measures, which may introduce common method variance. Future research could employ longitudinal or experimental designs to examine the dynamic interplay among MAQ, math anxiety, and teacher support, and to identify optimal timing for stage-specific educational interventions.

## Data Availability

The original contributions presented in the study are included in the article/supplementary material, further inquiries can be directed to the corresponding author.
